# Engaging multiple stakeholders to improve speech and language therapy services in schools: an appreciative inquiry-based study

**DOI:** 10.1186/s12913-019-4051-z

**Published:** 2019-04-15

**Authors:** A. L. Gallagher, CA. Murphy, P. F. Conway, A. Perry

**Affiliations:** 1 School of Allied Health, Faculty of Education & Health Sciences, University of Limerick, Limerick, V94 T9PX Ireland; 2 School of Education, Faculty of Education & Health Sciences, University of Limerick, Limerick, V94 T9PX Ireland

**Keywords:** Inter-professional collaboration, Health service improvement, Stakeholder involvement, Developmental language disorders, Child voice, Appreciative inquiry, Thematic analysis

## Abstract

**Background:**

Effective collaboration between speech and language therapists (SLTs) and teachers is essential in meeting the needs of children with developmental language disorders in school, but it is difficult to achieve. Currently, many children receive inadequate speech and language therapy services and/or support in school.

The aim of this study was to engage key stakeholders (SLTs, teachers, parents and children with DLD) in the co-design of their ideal speech and language therapy service and support in school. The study was undertaken in order to inform the development of a conceptual model to guide collaborative practice when working with this population.

**Methods:**

A qualitative study involving a diverse range of key stakeholders and using appreciative inquiry. This is a method which enables those involved to construct their ‘ideal’ about a topic of interest. Recruitment was carried out using purposive sampling. We conducted focus groups with practitioners (SLTs and teachers) and parents as well as semi-structured interviews with children who have DLD using ‘draw and tell’ techniques. A total of five focus groups and nine interviews were conducted with participants (*n* = 27).

**Results:**

The children described their ideal supports as those which enabled them to connect, contribute and achieve. They describe ways in which environmental barriers in school needed to be addressed to allow them to do so. The professionals primarily described ways in which the language skills of the child could be improved. Both parents and practitioner groups described the importance of strengthening networks between service providers and service users. They also highlighted the need to promote a collaborative culture if stakeholders are to work effectively together across sectors.

**Conclusions:**

There were differences in perspectives about the ways in which speech and language therapy services and supports could be improved, demonstrating the importance of engaging a diverse group of stakeholders. Of note were the unique insights the children brought about the barriers they faced as a result of their difficulties. Based on our findings we propose that children should be given influence in decisions about the supports that they receive in school. Implications for policy, research and practice are discussed.

## Background

For decades, inter-professional collaboration (IPC) has been recognised in policy as essential if outcomes for children with developmental disabilities in school are to improve [1–3]. This is particularly so for children with developmental language disorders (DLD) because their difficulties have implications - not just for communication in school, but for their learning [4, 5]. Working collaboratively in schools presents health service planners and practitioners with complex challenges, many of which remain unresolved. Consequently, many children with DLD go unidentified or receive inadequate support in school [6], resulting in poor long term outcomes. Specifically, children with DLD may do less well in national examinations [7, 8], are at risk of emotional behavioural difficulties [9, 10], can struggle to gain employment [11], and to live independently as adults [7].

According to the World Health Organisation [12], IPC occurs when, “two or more individuals from different professional backgrounds with complementary skills interact to create something that none had previously possessed or could have come to on their own” [p36]. The desired outcome of IPC is ‘collaborative advantage’ or the possibility of creating something greater collectively than that which is achieved when practitioners work alone [12, 13]. If SLTs and teachers can work collaboratively towards an agreed set of goals, then a child with DLD can attain improved language, literacy and educational outcomes [14–16]. In the process, practitioners may also develop enhanced skills and knowledge; that is, teachers may better modify their language to children with DLD in the classroom and SLTs may gain knowledge about the curriculum [17, 18].

### Theoretical framework of the study

D’Amour et al. [19] propose a model which provides a useful framework to develop our understanding of IPC in this context. The model has four elements, two of which relate to the process of collaboration at an *individual level* and two others to IPC factors at an *organisational level*.

Individual level dimensions are *shared goals and vision* and *internalization.* Having *shared goals and vision* refers to having an agreed set of outcomes and a direction to work towards. Internalization describes the degree to which those involved have an awareness of the differences between them, and the degree to which these differences are managed. According to D’Amour et al. [19], managing difference is necessary to foster a sense of belonging and of trust between across those involved. In the case of SLTs and teachers, several barriers at this level have been discussed in the literature. Some of these relate to professional/philosophical differences and others to practical/logistical issues. A lack of shared language and understanding between the professionals involved about DLD has been identified consistently as a barrier [20–22]. Further, many collaborative encounters between SLTs and teachers are ‘one off’, time-limited events, involving practitioners who are unfamiliar with one another. As practitioners don’t work together in a sustained way, it is difficult for them to develop an awareness of difference, and/or to develop the necessary trust and/or a sense of belonging [22, 23].

The two organisational dimensions include; *formalization*, the degree to which procedures exist that facilitate IPC (thereby clarifying expectations and responsibilities) and *governance* - leadership that gives direction to, and support for, collaborative working. It is difficult to determine the extent to which IPC is formalised between SLTs and teachers. In parts of the USA where SLTs are employed directly through education services, the school principal oversees the work of the SLT in school. However, it is not clear whether formal procedures exist at a school or a district level that relate specifically to collaborative planning and delivery of supports between SLTs and teachers. In the UK, Ireland and many European countries where SLTs are mainly employed by the health sector, formal cross-agency procedures to support IPC between practitioners are rare [24, 25]. In terms of governance, a recent review of speech and language therapy services in the UK showed continued variability in the extent to which school leadership supports IPC between SLT and teachers [25]. This is consistent with the findings of an Australian study where the need for leadership/organisational support for IPC has been identified in relation to work by SLTs in schools [26].

In summary, if SLTs are to be effective in meeting the needs of children with DLD in school, then they need to plan and deliver support collaboratively with the teacher. However, effective IPC is rare in practice with barriers evident, both at individual and at organisational levels. Our knowledge of how to facilitate IPC in this context is limited which can leave the child with DLD at a disadvantage, both socially and educationally.

In this paper we report the findings of the second of a multi-phased study aimed at developing a conceptual model to guide collaborative practice when working to meet the needs of children with DLD in school. In phase one, we examined the empirical and policy literature across the fields of speech and language therapy and education, searching for a shared understanding about this population that might inform the model. Whilst understanding perspectives in the literature is important, so too are the views of service users about how health services can be improved [27]. We therefore wanted to gain an understanding of what it is key stakeholders want from their ideal supports/services to schools.

### Aim, purpose and methodological approach

The aim of this study was for parents, SLTs, teachers and children with DLD to design their ideal speech and language therapy service and supports to schools. We were also interested in the degree to which the views of the different groups were aligned (or not) and the implications of this for successful IPC.

Given that the views of these stakeholder groups are relatively under-researched [28], we aimed to conduct our qualitative analysis inductively rather than with a pre-existing set of codes in mind in order to generate a rich description of the dataset as a whole. We conducted a thematic analysis at a semantic level, describing what participants said and interpreting this in relation to the previous literature, rather than undertaking a latent analysis, where the researcher is looking for meaning beyond what participants said [29].

We followed the ‘consolidated criteria for reporting qualitative research’ guidelines in reporting this study [30]. These ensure that sufficient detail is included in the reporting of a qualitative study to enable the reader/reviewer to appraise the quality and rigour of the research.

## Methods

### Participants

A purposeful sample of participants was recruited to the study. The sample included 29 participants in total: SLTs (*N* = 8), teachers (*N* = 5), parents (*N* = 9) and children with DLD (*N* = 7). Each professional was recruited considering their current post, years of experience, gender, and either work setting (SLTs) or type of school in which they work (teachers) for wide representation. Children were recruited according to age, gender and primary diagnosis, as well as different types of speech and language therapy support received and type of school attended. Parents (fathers and mothers) were recruited across Ireland and came from different socio-demographic backgrounds. Collectively, they had experience of accessing the full range of speech and language therapy services and supports currently available for children with DLD in Ireland. Parents and children were recruited via a national support network for parents with DLD using snowballing techniques and practitioners were recruited through professional bodies and established clinical networks via email/phone contact. See Participant details in the Results section.

### Topic guides

We used appreciative inquiry when developing the topic guides for the focus groups and interviews. Appreciative inquiry was developed by Cooperrider and Srivastva [31] in the field of organisational psychology as a method of generating new ideas about a topic of inquiry. The approach does not start with a pre-defined ‘problem’ that needs to be fully understood in order to remediate it, but enables those involved in the process to focus on the ‘ideal’ situation. It has been previously used successfully to document the views of children who have DLD about how they would like their life to be in the future [32]. A pilot session with one SLT, one teacher and a parent of a child with speech, language and communication needs was run, to refine the topic guide for the focus groups. The activities were piloted with two children also prior to conducting the interview (see Appendices 1 and 2 for topic guides).

### Procedure

#### Focus groups

**Fig. 1:**
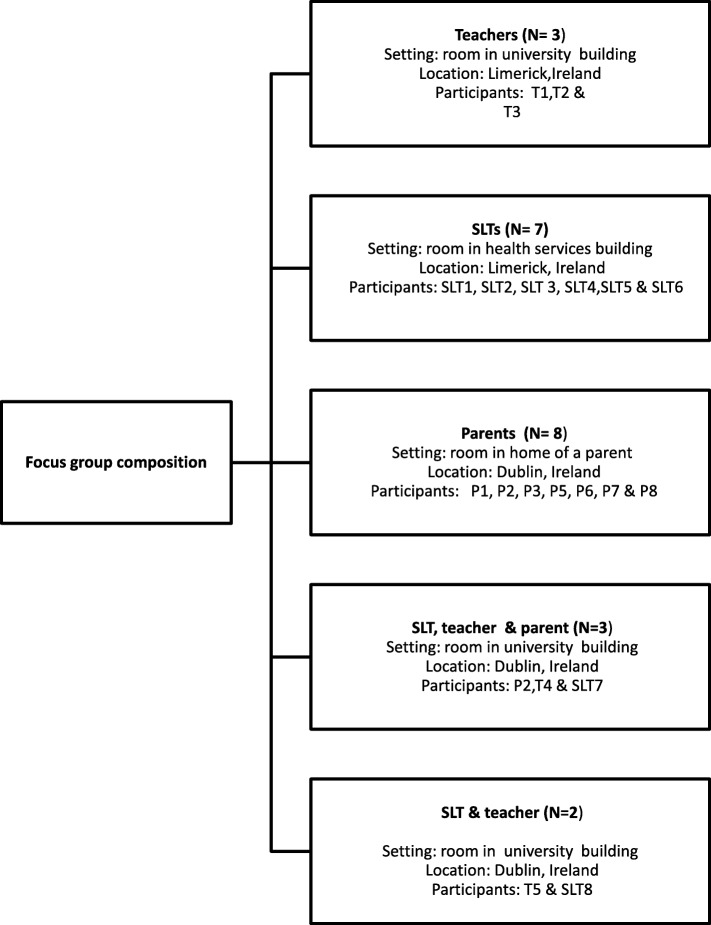
Composition of focus groups with speech and language therapists, teachers and parents

Five focus groups were held with practitioners and parents. These included three same-participant groups and two mixed participant groups. It was planned to have two mixed groups with all three participants (SLT, teacher and parents). However as some parents did not wish to attend such mixed groups, only one group had all three participant types; a second was attended by one SLT and one teacher (see Fig. 1 for a summary of groups held, location and participants involved).

All focus groups were facilitated by the first author (ALG), a PhD candidate and qualified SLT who had worked previously with school-aged children with DLD and had undergone additional training in appreciative inquiry. The parents, teachers and children who participated were not known to the SLT prior to the study. Two of the SLTs who took part were known to ALG in a professional capacity from attendance at professional forums.

The participants were informed in writing of the aims of the study and the professional background of the facilitator prior to gaining consent. The facilitator had further phone/ email contact about the study prior to data collection with the participants. At the beginning of each focus group, the facilitator introduced herself, described her prior clinical experience and interest in working in schools, and the aims of the study. The focus groups each lasted between 60 and 70 min.

An observer was present at each session to document any non-verbal interactions and/or actions that occurred between participants and the facilitator, using a standard observation checklist. The observers were PhD candidates currently undertaking qualitative research projects. Following each focus group, a discussion was held between the observer and facilitator about these observations, with the discussions audio-recorded for later integration with the transcripts during analysis.

#### Semi-structured interviews

Seven semi-structured interviews were held with the children. The children were given the choice to be interviewed alone or with someone else present. Two children were interviewed with a parent present and one with their sibling. The facilitator met the children on two occasions. The purpose of the study was explained at the first occasion; planned activities were demonstrated and the participant(s) became familiar with the facilitator. The interview proper was then conducted at a second visit. The facilitator recorded field notes directly after each interview.

When planning the interviews for children with DLD, consideration was given to issues of participation, trust, consent, power, and control [33]. For example, each child was given a red and yellow card at the start of the group. The children knew that they could show the facilitator the yellow card if/when they struggled to understand a task. This signalled to the facilitator the need to adapt or simplify her language. All but one child used this strategy during the interviews. They also knew that they could withdraw their consent to participate at any time, by raising a red card. Draw-and-tell techniques were used with the children. This widely-used technique encourages children to participate by reducing the pressure on a child to communicate verbally [34, 35]. The children’s comprehension of tasks was assisted by employing augmentative methods of communication. The duration of the interviews was influenced by the communication abilities of the children, varying between 35 and 50 min. All interviews were audio-recorded.

### Data transcription and analysis

There were three researchers directly involved in the transcription and analysis (author 1 and two researchers). The interviews were transcribed post hoc by author 1, also the facilitator of the groups. These were checked for accuracy by researcher 2. We followed Braun and Clarke’s six-phased guide when analysing the data [29]. Data were managed using NVivo 11©, a software package which enables a large amount of coded text to be sorted and tracked and for analytical notes about coding decisions to be stored. This enhanced transparency in analysis.

### Coding

A transcription from one of the focus groups was randomly selected by researcher 2 for double-coding. A section of this transcription was coded by each researcher independently, and coding decisions were discussed. When both researchers felt there was, “consistency of meaning” [36] - viz., there were few differences evident in relation to the coding decisions – a further section was coded in the same way. This process was undertaken for the transcripts from the semi-structure interviews also. In total, one full transcript from the focus groups and two transcriptions from the interviews were coded in this way. A process of constant comparison was undertaken to generate codes until a final set of codes was identified. Researcher 3 then examined the codes that had been generated from the data and made suggestions about merging some of them. From this, categories were generated which were descriptive, rather than interpretive. Once the codes were organised into categories, key themes were identified. These were presented to co-authors (CAM, PC and AP) on three occasions for refinement. Finally, the themes were re-presented to the participants for checking/comment.

## Results

### Participant details

Details of the participants involved in the study are set out in Tables 1, 2 and 3 below.

**Table 1:** Participant details (professionals)

Participant reference	M/F	Current role	Employer	Work setting	Professional experience
T1	F	Class teacher	Education	Primary school^a^ (DEIS^b^)	4 years
T2	F	Class teacher	Education	Primary school	6 years
T3	F	Resource teacher^c^	Education	Primary school (DEIS)	6 years
T4	F	Resource teacher	Education	Language class^d^	3.5 years
T5	M	Principal	Health	Primary school	10 years
SLT1	F	SLT	Education	Secondary school	4 years
SLT2	F	SLT	Health	Primary care^e^	8 years
SLT3	F	SLT	Health	Primary care	1 year
SLT4	F	SLT Manager	Health	Primary care	11 years
SLT5	M	SLT	Health	Primary care	7.5 years
SLT6	F	SLT	Health	Primary care	2.5 years
SLT7	M	SLT	Health	Language class	2 years
SLT8	F	SLT	Education	Secondary school	9 years

*T* teacher, *SLT* speech and language therapist

^a^In Ireland, children attend primary school from the ages of five to twelve and secondary school from the age of 12 to 18

^b^DEIS is an acronym for ‘Delivering Equality of Opportunity in Schools.’ It is a category of school within the Irish education system which serves a population of high social need, and is allocated additional resources

^c^resource teacher is a teacher specifically responsible for the delivering of additional support for children with special education needs in schools in Ireland

^d^a ‘special’ class with reduced numbers of children in a mainstream school, all of whom have severe DLD. There is an SLT (employed from the local health service) assigned to the class, providing regular input

^e^SLT provided in the community as part of a primary care team

**Table 2:** Participant details (parents)

Participant reference	Relationship to child	Location	Speech and language therapy services accessed
P1	Mother	Dublin	Primary care service^a^, language class^b^ & CAMHS^c^
P2	Mother	Limerick	Primary care service, language class & private SLT
P3	Mother	Dublin	Primary care service, language class & special school
P4	Mother	Clare	Primary care service
P5	Mother	Cork	Primary care service
P6	Mother	Dublin	Primary care service & private SLT
P7	Mother	Dublin	Primary care service
P8	Father	Dublin	Primary care service
P9	Father	Tipperary	Early intervention^d^, primary care service & language class

*P* parent

^a^speech and language therapy service provided in the community as part of a primary care team

^b^a ‘special’ class with reduced numbers of children in a mainstream school, all of whom have severe DLD. There is an SLT (employed from the local health service) assigned to the class, providing regular input

^c^Child and Adolescent Mental Health Services

^d^multi-disciplinary team of health professionals who provide diagnostic services and treatment for children with multiple needs prior to school-age

**Table 3:** Participant details (children)

Participant reference	Gender	Age	Type of provision	School type Urban/Rural	Diagnosis
C1	M	12	Mainstream	Urban	DLD^a^
C2	F	11	Special^b^	Rural	DLD
C3	M	13	Mainstream	Urban (DEIS^c^)	DLD
C4	M	12	Mainstream	Urban (DEIS)	DLD
C5	F	11	Mainstream	Rural (DEIS)	DLD
C6	M	10	Mainstream	Urban	DLD
C7	M	13	Mainstream	Rural (DEIS)	DLD &EBD^d^

*C* child

*DLD* ^a^developmental language disorder

^b^a school catering exclusively for children with additional needs

^c^DEIS is an acronym for ‘Delivering Equality of Opportunity in Schools.’ It is a category of school within the Irish education system which serves a population of high social need, and allocated additional resources

^d^Emotional behaviour disorder

### Themes

**Fig. 2:**
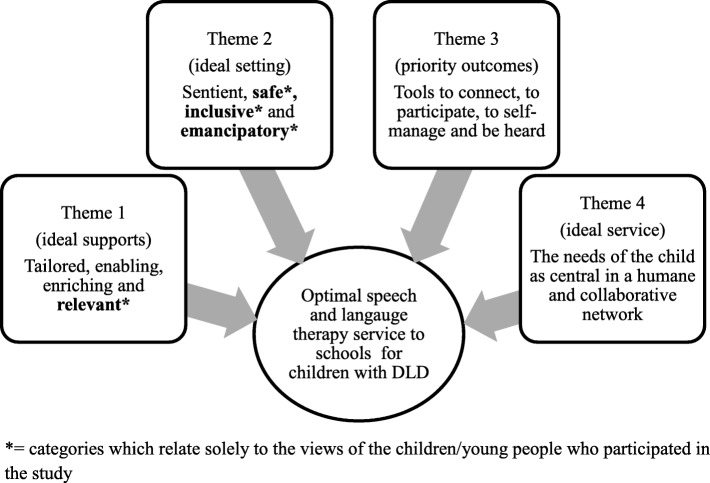
Overview of themes

Four themes were identified in the dataset (see Fig. 2 for an overview of the themes). These related to: (i) the nature of the ideal supports for the child with DLD in school; (ii) the ideal setting; (iii) desired outcomes for the child with DLD and (iv) characteristics of the ideal service.

In Table 4, we present the supporting categories that map the contributions of each stakeholder group to each theme. Examples of direct quotes from the dataset are provided under each theme. In these quotes, we have a used a process of ellipsis to improve readability. This involves replacing fillers/hesitations using a series of dots. Quotes have not been edited beyond this process.

- **Theme 1:** *The ideal supports are tailored, enabling and enriching, and enhance the child’s learning and social capital*

**Table 4:** Themes and supporting categories

Theme	Supporting categories
*1. Tailored, enabling, enriching and relevant supports that enhance the child’s learning and social capital*	a. Tailored (individualised^a^, proactive^b^) b. Enabling (strengths-based^a^, supports independence^a^, supports the ability to make choices^a^) c. Enriching^a^(more than language^a^, beyond the classroom^a^) d. Relevant^c^ (supports that address issues of inclusion and exclusion^c^ and the development of social capital^c^)
*2. A sentient, safe, inclusive, emancipatory setting*	a. Sentient (listening^b^, noticing^b^) b. Safe^c^ (to make mistakes^c^, explicit expectations^c^, consistent responses^c^) c. Inclusive (diversity as valued^c^, presuming competence^c^) d. Emancipatory (democratic^c^, power and control^c^)
*3. To be able to connect, participate, self-manage and be heard*	a. Connect & participate^a^ (friendship skills^a^, negotiating entry^c d^ contributing in class^c^) b. Understand (people^c d^, rules^c^) c. Self- manage (awareness of own needs and feelings, able to self-regulate^d^, able to seek support^b^, strategies for survival^c^) d. Have a voice (speaking up & speaking out^d^, influencing^d^)
*4. The needs of the child as central in a humane and collaborative network*	a. Needs-led (aims^b^, resource allocation^b^) b. Humane (ethic of caring^b^) c. Collaborative (equality in relationships^b^,shared responsibility^b^) e. Network: (professional autonomy^b^, responsive^b^, blurred roles and boundaries^d^)

^a^views expressed by all stakeholders

^b^views expressed by parents, SLTs & teacher

^c^views expressed by children only

^d^views expressed by parents only

SLTs, teachers, parents and children described ideal supports as being *individually-tailored* to their/ a child’s /needs and interests. These supports were described, not as prescribed procedures, but as supports that are regularly reviewed and refined, according to an individual child’s changing needs (Table 5, quote 1).

**Table 5:** Quotes from the dataset related to theme 1

Quote No.	Participant reference	Quote
1	T1	“… you do it for a certain amount of weeks but then you need to change it up again depending on the child’s response … every child responds differently to what’s done in class, it is the response that counts”
2.	P9	“supports … so they (the child) are not overwhelmed but that the learning is set just right for them, cos they know the child so well and then the child can succeed”
3.	C5	“I can do the work it’s hard but not too hard … so I’m learning … but I feel good”
4.	T4	“… finding out things that motivate him or topics he likes talking about and starting from there”
5.	C4	“... I like learning about the past. I want help with hard stuff that I like, like that”
6.	T3	“… to find a hidden talent or an activity for him that he’s good at … then use that learning to help him with other activities …”
7.	T2	“… so the supports allow him to take risks and have a go at things himself”
8.	P2	“… if he could be supported to get better at problem solving – great but then to practise this, now that would be good support”
9.	P1	“… they have to be practised … he needs to be given the language to do it and loads of chances to practise it”
10.	C1	“I want to pick books myself to read...help to choose a good book for me, not just what’s the reader, at school, we just do the readers”
11.	C1	“I like experiments it makes it easy to learn if you are doing it. So we did an experiment before with washing up liquid and more art cos you can think about things, it’s another, it gives you another way to think about things”
12.	P2	“They went to the fire station and afterwards, it’s what they did with it – they didn’t go into books, they talked about the day, so they were using the words and practising communicating with each other”
13.	T4	“(For the child with DLD) it’s so important getting away from books and do things in a real life context”
14.	P7	“if the SLT went in to see the dynamic in there (the classroom), they might be able to help with how things are done … that would really help him every day”
15.	T2	“it’s about crossing context … the home as well as the school … supports need to continue in the home as well …”
16.	C3	“Yeah the rules just don’t make sense and also sometimes they (the teachers) say don’t have a phone in school and but they (children) do have a phone in school and they (the teachers) know it. I don’t get it … I want real help with understanding the rules that can be broken”
17.	C2	“I want to be cool... for others to think I’m cool … so they will want to play with me … can someone learn me that?”

*C* child, *P* parent, *SLT* speech and language therapist, *T* teacher

Tasks in the classroom would be set at the ‘right level’ of difficulty, with the ‘right amount’ of scaffolding, so that each child would be challenged (Table 5, quote 2). One of the children described positive feelings when this balance of being challenged but supported was achieved previously with regard to their learning (Table 5, quote 3).

Supports which capitalise on the *interests* of the individual child were discussed by all – teachers (Table 5, quote 4), parents and children. The children for example, referred to support that helped them in the subjects that interested them (Table 5, quote 5).

A further characteristic related to support which is *enabling* that is, delivered in a way which makes a child feel that they can succeed. Teachers discussed the idea of a strengths-based approach to support a child in school (Table 5, quote 6). This involved knowing what a child can do and then using this knowledge to facilitate success in tasks that are difficult for them.

Support that provides a child with a set of tools to use in different situations was also discussed. Such tools would enable a child to become more independent in classroom learning (Table 5, quote 7). Parents were clear, however, that in order to get the child to be able to use such tools, they would need to be explicitly taught to do so, in the relevant context by practitioners (Table 5, quote 8 & 9).

Support that enables the child to make informed choices about their learning was discussed by children and parents. One child for example, described having support to make good choices about books; books that would help them to become better at reading (Table 5, quote 10).

A third characteristic related to the importance of providing *enriching* learning opportunities that are not delivered through language instruction alone. These may occur in the classroom (Table 5, quote 11) as well as outside of school. One parent describes a trip out of school, which they felt was effective in supporting language learning (Table 5, quote 12). One teacher explained the idea of “active learning,” which she felt would be an effective approach for planning support for a child with DLD (Table 5, quote 13). Parents discussed the importance of practitioners working across contexts in delivering supports. They described the SLT working in the classroom with the teacher (Table 5, quote 14). Teachers discuss the need for home and school to work together to consolidate learning (Table 5, quote 15).

A final characteristic, described by the children only, related to support that was *relevant* – that is, informed by the children’s experiences of times when they are excluded socially or unable to participate in school. Their ideal supports would provide them with the skills to navigate the ambiguities of social contexts and relationships and which enabled them to contribute in class.

One child, for example, expressed the desire for help to decode the ‘unspoken rules’ in school, which he described as those that were understood by others -teachers and children- but not by him (Table 5, quote 16). Another child talked about the possibility of having assistance to learn how to enhance her social status among her peers, so that she would be included (Table 5, quote 17).

- **Theme 2:** *The ideal setting is one which is sentient, safe, inclusive and emancipatory*

Of all the stakeholder groups, it was the children who contributed the most to this theme.

Parents and practitioners described their ideal classroom setting as sentient;-that is, one where there was a culture of listening and noticing. In their ideal classroom all those involved with the child with DLD would make the ‘effort’ to listen to them (Table 6, quote 1). If the child was not engaged in learning, this would also be noticed, and the child would be encouraged to do so (Table 6, quote 2).

**Table 6:** Quotes from the dataset related to theme 2

Quote No.	Participant reference	Quote
1	T2	“(In this school), he gets the feeling that people have time for him. And can be bothered to figure out what it is he wants to say …”
2.	P8	“he (the child with DLD) needs someone to notice him … and to coax him out and to encourage him to try”
3	C3	“not so much pressure to answer questions … instead of asking us quickly for the answer … and looking stupid”
4.	C5	“in this school if you are mean you have to go to time out no excuses”
5.	P7	“To be accepted …. I think all of us want our child to be accepted. I should add- easily. I mean all the other kids are accepted easily aren’t they?”
6.	C2	“so all these students are very different … there is all sorts in there … all with different talents”
7.	C3	“They (the students in the ideal classroom) wouldn’t say ‘I’m better than you’ … they say ‘everyone is different’, ‘you’re good at this’ and ‘he is good at that’”
8.	C2	“they (the students) like each other, because mmm.. normally people don’t like them because they are very strange and they just don’t like them ...but strange is good in this school”
9.	C4	“Yeah, the kids are in charge in my school … they decide …”
10.	C1	“The teacher does all the talking and the children are not allowed talk- in this class the children can talk”
11.	C3	“… the teacher is always asking us for the answer … it annoys me the way the teacher asks a question that they know and you might not know it and you have to say ‘I don’t know’ and you act like a fool”
12.	C1	“In class … there is more time to talk... and more chances to practise talking cos... it helps you think about things”

*C* child, *P* parent, *SLT* speech and language therapist, *T* teacher

The children spent most of their time talking about their ideal setting. They described it as one, which is *safe*, *inclusive* and *emancipatory*. They described a classroom in which they felt *safe* to take risks with their learning and talking, without fear of negative exposure (Table 6, quote 3). There would be clear and explicit expectations (aka rules) about how students treat each other in this classroom (positively, and with respect for each other) and there would be consistent responses from teachers to reinforce these values (Table 6 quote 4). This characteristic was discussed when the children recounted experiences of being bullied and/or where they reported feeling exposed and humiliated in a class.

All children described a setting which was *inclusive.* While parents and practitioners focussed on the importance of the child with DLD being accepted (despite their differences) by the rest of the children in their peer group (Table 6 quote 5), the children described a classroom where *every* child is seen as different (Table 6 quote 6). In the children’s ideal setting, difference would be openly discussed and celebrated as a positive resource (Table 6 quote 7) and the children would like each other *because* of their differences, rather than despite them (Table 6 quotes 8).

A final characteristic of their ideal setting, discussed by the children, related to *power* and *control*. Their ideal setting was one where the children had influence in decisions (Table 6 quote 9), and where they had more control over speaking in the class (Table 6, quote 10).

All of the children talked about how language is used and by whom, in the classroom. In their ideal classroom, they would be given the space to use language for a variety of purposes rather than being restricted to answering the teacher’s questions. This was in the context of recounting feelings of humiliation due to being unable to provide the right information requested by the teacher, in front of their peers (Table 6 quote 11). In their ideal classroom, children would have more control over how language can be used and more opportunities to use language to think (Table 6 quote 12). The children were clear that they need to be given more chance to practise talking, for their language to improve.

- **Theme 3:** *The child with DLD will be able to connect and participate, understand, self-manage and have a voice in their lives*

All the stakeholder groups mentioned the ability to connect with others and to maintain good quality friendships as a priority skill for children with DLD to learn. Whilst practitioners and parents discussed the outcome of ‘having a friend’ in broad terms (Table 7, quote 1 & 2), the children described the skills they need to be able to achieve this, specific to the context of the nature of social relationships that are formed and maintained in school. For example, one child discussed needing to *learn the language of their peers* as a ‘way in’ (Table 7, quote 3).

**Table 7:** Quotes from the dataset related to theme 3

Quote No.	Participant reference	Quote
1	T5	“to have good friends., true friends”
2.	P6	“they are not in the clique – in the gang, they are outsiders and don’t know how to get in- they need to know how to get in”
3.	C1	“I want to talk, you know like, talking the way they (peers) do, so they will listen and think I’m interesting”
4.	SLT7	“he is putting his hand up in class.. participating … he knows the answer”
5.	C1	“to be able to talk more in class, so I can to try out new ideas”
6.	P6	“they need to be able to figure out the grey areas, you know, reading other people’s intentions”
7.	C2	“… to be able to listen to people’s thoughts and see inside their head”
8.	C6	“if this person was feeling this way … knowing how that person is feeling … learning what would you do”
9.	T1	“everybody has strengths and weaknesses. The important thing is that you know yourself so you can help yourself”
10.	P6	“I want him to be able to notice that (how he feels) himself and be able to do what he needs to keep himself right”
11.	SLT8	“I think for children who don’t understand, it would be one of the key strategies to actually know it and say when they don’t understand”
12.	C2	“… everybody thinks he is really brave but inside he is a really scared guy, he just acts like a tough guy in front of people … and they believe him and they leave him alone”
13.	P7	“to be able to get stuck in and fight his corner in there- in a good way obviously”
14.	P9	“to express himself when he feels it’s not fair in school”
15.	P6	“life is about choices and decisions – nothing is black and white, everything can be negotiated...you can shape your own choices … I want him to be able to do that”

*C* child, *P* parent, *SLT* speech and language therapist, *T* teacher

All stakeholders stated that being able to participate in class was an important outcome but there were diffe rences in perspective about the meaning of participation. For teachers and SLTs, successful participation was described as the child with DLD being able to demonstrate the required knowledge in ‘typical,’ tightly-controlled classroom interactions. They repeatedly described a child with DLD with their hand up, willing and being able to answer a teacher’s question (Table 7, quote 4).

The children, by contrast, wanted to be able to participate, not to demonstrate knowledge but rather to contribute to the development of ideas (Table 7, quote 5). For the children, participation was discussed as a means by which they could learn through language.

Another outcome, described by both parents and children, was the ability to understand or, more specifically, to make inferences about people and social situations (Table 7, quote 6). One child stated that, if they were a super-hero, their special power would be ‘to be able read people’s minds’ (Table 7, quote 7). Another child discussed their wish to be able to ‘read’ other children and to know how to respond in a suitable way (Table 7, quote 8).

Three children said they wanted to understand the ‘unwritten rules’ in school, which they struggled to comprehend. They discussed this outcome in the context of describing how their current inability to do so, resulted in their being excluded from the school community.

A further outcome related to a child with DLD being able to independently manage their needs in school. All stakeholders discussed the importance of self-management, although they had differing views about the purpose of having such skills. Parents and professionals talked about self-management in the classroom only. They wanted a child with DLD to be able to use strategies to manage their learning and language needs. They discussed the importance of knowing when to seek support (Table 7, quote 9 and being able to regulate feelings and behaviour in readiness for learning (Table 7, quote 10).

SLTs, in particular, emphasised the importance of a child being aware of their comprehension difficulties in the classroom and being able to signal to the teacher when they needed help in understanding (Table 7, quote 11).

The children wanted strategies to manage complex/nuanced issues related to navigating ethical dilemmas and peer relations. They also talked about the need for strategies that would help them to ‘survive’ and ‘stay safe’ in the context of their relationships with peers. For example, one child described developing an outer personality (which was contradictory to how they felt inside), to avoid being a target of bullying (Table 7, quote 12).

A final outcome, mainly discussed by parents, related to children with DLD ‘having a voice.’ They wanted the child with DLD to be able to stand up for her/himself (Table 7, quote 13) and to speak up when they encountered injustice (Table 7, quote 14). Parents wanted their children with DLD to be able to influence those around them in decisions, which impacted on their lives (Table 7, quote 15).

- **Theme 4:** *The ideal service is humane and collaborative and places the needs of the child with DLD as central*

Parents and practitioners described a service, which should be, first and foremost, ‘needs-led’. In other words, all of the service and resource decisions (who is involved with the child and family, for how long, and in what way) would be underpinned by one agenda - the child needs (Table 8, quote 1) and to deliver these (Table 8, quote 2). This is in contrast with parent’s perceptions of current speech and language therapy services, which they perceived to focus on *limiting* the child’s access to resources.

**Table 8:** Quotes from the dataset related to theme 4

Quote No.	Participant reference	Quote
1.	P1	“(the service) it is child-centred so it’s a service where they don’t care about resources or what they’re entitled to, no, they will push the boat out”
2.	SLT	“… more solutions … more actual support … so much time is spent finding out what is wrong instead of trying things out that might help”
3.	SLT5	“…. this service isn’t restricted to his language only but his ability to interact more broadly”
4.	SLT4	“the service also helps to adapt the environment he is in … so that he can learn”
5.	P2	“What about an infinity symbol? … it’s like a figure of 8 or something … continuity and no break in services”
6.	P2	“So it’s these arms … that hug that says, we’re there for you, we’re reaching you, we care about you”
7.	SLT6	“The hand is for helping and a circle all around him (the child). There’s lots of people in his circle, they care”
8.	P9	“A listening service ... a service that listens to you and respects you as a parent. Parent opinions are heard … everyone has something to bring to the table and everyone’s input needs to be respected equally”
9.	SLT1	“I am putting an ear so that he (the child) is listened to and a speech bubble so he has a voice”
10.	T5	“they (decisions) are led by child and parent … not the school or the professionals. So they identify what are the difficulties and they decide together how the people should address those difficulties”
11	P1	“everything has been a battle from day one. It’s affected my mental health. It has worn me down”
12.	T5	“The SLT … I want her in … no … inside the inner circle not just in and out but actually getting stuck in to the goings on day to day … sharing the load in the classroom”
13.	P7	“I just want the SLT in the school. I just want them in.. to be part of it- get stuck in.. helping in there...”
14.	SLT1	“Not a top down service no.. not that … not ‘it is not our policy’ or ‘oh you’ve actually gone above what you’re entitled to’ … or ‘this is what we do’ … more than that … the practitioner being able to decide, cos you know the person best”
15.	P8	“there is no waiting and the service follows the kid. Like early intervention … the difference is huge …. so not waiting and seeing.. but getting in there quickly”
16.	SLT7	“..things are continually changing …. like the new oral language curriculum... the service needs to be able to respond to new developments all the time”
17.	P7	“the service allows the practitioner to be part of it- get stuck in and help him (the child) to fight his corner in there (in school)- in a good way obviously”
18.	P4	“You can’t learn that (real life skills) in a clinic room.. no way. So in this service she (the practitioner) is able to get really getting messy with it and get into the nitty-gritty with the child …”

*P* parent, *SLT* speech and language therapist, *T* teacher

The ideal service, according to parents and practitioners, considers the classroom environment when determining the needs of the child. The focus of interventions and the outcomes which are measured by such a service are not just related to clinical outcomes, but also to social interaction (Table 8, quote 3 & 4). Parents, in particular, highlighted that the ideal service would address the barriers the child faces in school because of their language difficulties.

The ideal service, according to the parents, offers continuity of care as it is also informed about the persistent nature of a language disorder, and aware that it is a lifelong condition (Table 8, quote 5). They discussed the negative consequences for a child and family of being moved, ‘in and out’ of different services, which they had experienced previously.

A second characteristic was that an ideal service would have, at its core, an ethic of care for the service users as well as for those who deliver the service. When representing this service on paper, parents, SLTs and teachers frequently drew arms and/or a heart around a child to emphasise that they would be ‘cared for’ within an ideal Speech and language therapy service (Table 8, quote 6 &7).

A third characteristic related to the collaborative nature of the ideal service. Collaboration was described as involving two key elements; *equal* partnerships and a *sharing* of responsibility.

Equal partnerships were described as those where parents and their children with DLD were listened to; where their opinions counted (Table 8, quote 8 & 9). Parents and practitioners described how decision-making about supports would be shared between the parent(s), practitioners and the child (Table 8 quote 10), rather than (as at present) being controlled by the professional(s) alone.

A sharing of responsibility in meeting the needs of the child with DLD in school by all those involved was discussed by all stakeholders.

Parents discussed it in the context of recounting feelings of exhaustion from being left to co-ordinate (and fight for) supports for their child on their own (Table 7 quote 11). Teachers and SLTs discussed it in recounting strong feelings of frustration about current service models where children with DLD were described as falling through the gaps.

The stakeholders differed in their views of the role of the SLT in their ideal service. The teachers were very clear that they wanted the SLT physically present and working in the classroom, whereas the SLTs positioned themselves more as ‘advisors.’

In one group, when the SLT and teacher drew a picture of their ideal service and those involved, the teacher insisted that the SLT be moved to the “inner” circle (Table 8, quote 12). (where the group had positioned the teacher and child) from an outer one, where the SLT had initially placed herself. The teacher made a clear distinction between Speech and language therapy services where the therapist was minimally involved in a ‘consultative’ role - and their ideal service, where the SLT as a *true* collaborator ‘on the ground.’

The parent’s views were aligned with those of the teachers in how responsibility for their child could be shared in their ideal service. They also wanted the SLT to be *in the school* and *working in the classroom* (Table 8, quote 13). Frustration was expressed by parents and teachers at current ways of working, where parents stated that SLTs ‘passed on their responsibility’ for a child’s language development to school staff.

A final characteristic described by both parents and practitioners related to the values of the ideal organisation in which the service sits. An SLT described an organisation in which the clinical expertise of practitioners is recognised and where they are given the authority to make decisions and to act in the best interest of the child (Table 8, quote 14).

In this ideal organisation, those providing the service can *respond* easily and quickly to the needs of the service users, as well as to external influences, such as new research findings and/or policy changes (Table 8, quote 15 & 16).

This ideal organisation is focused on relationships between people and strengthening these. Stakeholders were clear that supporting strong relationships across sectors is required if practitioners are to make collaborative decisions, in the best interest of the child. In this ideal service, practitioners would be supported to work beyond the traditional boundaries of an SLT in a health clinic. This would allow the practitioner to meet the needs of the child in the context of their everyday life in school (Table 8, quote 17 & 18).

## Discussion

The aim of this study was to characterise the views of multiple groups of stakeholders when asked to describe their ideal speech and language therapy service for children with DLD in school. We identified convergent and divergent views, within and across the participant groups, about services and supports. We discuss the implications of our findings with reference to the four elements of IPC as described by D’Amour- shared goals and vision; internalization; formalization; and governance [19].

The goals of the children differed from those of the practitioners. They were primarily concerned with their social inclusion and participation in school, consistent with previous studies [37, 38]. They described many of the barriers that they faced as a result of their language difficulties on a daily basis; barriers such as the different registers of language used by peers, the ‘hidden curriculum’ through which values, norms and rules in school are tacitly transmitted, and the restrictive rules about how language can be used in the classroom.

The priority goals of the children were to facilitate their inclusion, participation and achievement. This goal cannot be achieved solely by equipping them with the necessary languages skills and tools, but also requires environmental barriers to be addressed. They talked, for example, of support that would help them to learn to speak the language of their peers, and those supports which would help them to understand the implicit rules of the school, as well as the need to create opportunities for them to use language in class for thinking. In contrast, the main goal of supports from the point of view of the practitioners was to improve the language skills of the child. They did not discuss the ways in which the classroom and/or school setting might enable or disable a child with DLD.

There were also differences between the children and practitioners in terms of their vision of the ideal classroom and school. The children provided a clear picture of their ideal classroom setting as one which is inclusive. In this inclusive setting, all children were acknowledged to be different, diversity was celebrated, and children were liked *because* of their differences, rather than *despite* them. The children also described their ideal classroom setting as one where they were given the autonomy to make choices about their learning and where they were enabled to participate*.* For them, participation meant being able to contribute to the co-production of ideas. They described ways in which practices in the classroom could be adapted to enable them to do so, such as having a less restrictive classroom discourse. While practitioners did refer to such principles as inclusion, autonomy and participation, they struggled to create a vision of an inclusive classroom and school. For example, having stated the importance of an inclusive setting, they went on to describe their ideal classroom as one in which the child with DLD (who is different to the other children) is successfully integrated;-that is, accepted despite their differences. Similarly, while acknowledging the importance of the child with DLD having autonomy in principle, they did not discuss ways of adapting the classroom and/or teaching so that the child could actually exercise choice. Likewise, whilst discussing the importance of a child being able to participate in school, practitioners did not describe ways in which classroom discourse might be adapted in order to facilitate this. These findings suggest, consistent with the literature, that practitioners may not have a clear understanding of how inclusive principles might be enacted [39].

The differences relating to the goals and vision might be due to where the different stakeholder groups ‘*rest their gaze.’* According to Henderson, cited in Graham [40], the cause for failing to learn certain skills can be understood in different ways; - viz, due to the ‘deficient child’ or the ‘deficient teacher’ [p.10]. Practitioners in this study focused mainly on how support and services can improve the skills of the ‘deficient child’, whereas the children were primarily concerned with the ways in which the environment/ practices is deficient and could be adapted to facilitate their participation and inclusion in school. These stakeholders appear to have a different understanding of both ‘the problem’ and ‘the solution’ in relation to how best children’s language needs can be met in school.

Tangen [41] offers an explanation for the differing perspectives we identified in this study. She discusses the concept of ‘insider’ knowledge - that which can be gained only through direct experience. The children with DLD in this study brought their unique insider knowledge about the barriers they face in school as a result of their difficulties; yet children with DLD are not routinely included in decisions made about supports to be delivered to them in school [42]. The findings of this study show that omitting to include the perspective of the child may result in barriers to their participation and achievement and/or potentially discriminatory practices to remain unchecked.

Professional differences have been discussed in the literature as a barrier to collaboration between SLTs and teachers [19–21], as well as between professionals and parents [43]. Including the child in decision-making would add further differences in perspective, and power issues related to the status of the child relative to the adults. This requires very careful consideration about how such differences could be acknowledged and managed;- that is, how such differences could be ‘internalised.’

It is important to highlight the agreement we found between all stakeholders about the *nature* of the ideal supports. All participants described *s*upports which are individually-tailored, enabling and varied. These views are consistent with those of parents reported by Roulstone [28] and of teachers, reported by Dockrell et al. [44] when describing speech and language therapy services in the UK. The importance of strategies that enable a child to become a more independent learner has also been previously documented [32, 45]. Such agreement has positive implications for the collaborative process, provided that a set of shared goals and a collective vision can be agreed and differences are managed.

Parents and practitioners were closely aligned in their views when describing their ideal service. They discussed a service in which the quality of relationships is central and where there is an ethic of caring. They characterised their ideal service as a series of collaborative networks which include the service user (parent and child) and service providers (SLTs and teachers). They described collaborative relationships as those in which there is equality and shared responsibility. Shared responsibility, for these stakeholders, meant everyone having a role not just in the planning but in the delivery supports in school and in the classroom.

Formalization of processes and procedures by setting up and strengthening such networks between SLTs, teachers and stakeholders may be a way of improving the quality and effectiveness of collaborative services to schools. Several case studies have been reported that describe different methods of strengthening such networks and the role of leadership in doing so [46].

Parents and practitioners also describe a service, which is responsive, flexible and innovative. These characteristics point to a particular organisational ‘culture’ or set of values, referred to in the literature as ‘adhocratic’. This culture, according to Ovseiko et al. [47] promotes adaptability and risk-taking at the ‘ground’ level, and is distinguished from a ‘bureaucratic’ one where decisions are made at the ‘top,’ to which workers must adhere.

Historically, attempts at enhancing collaboration between speech and language therapy services and schools have focused on reducing only the structural barriers, without considering cultural factors. In terms of governance then, leadership which promotes such a culture may be warranted and there are tools such as the ‘Competing Values Framework,’ piloted across a wide range of organisational contexts, which could guide SLT managers and school principals in doing this [48].

In summary, the findings of this study show the benefits, not just of including diverse groups of stakeholders in health services research to inform service improvement but also of including these different perspectives in an everyday capacity, when planning the delivery of speech and language therapy supports in school. Parents.

### Implications for policy

We propose that a key policy implication of the study across health and education is to reinforce the status of the child as a ‘being’ in their own right. This is necessary so that including the child/young person in decisions is a requirement rather than desirable/conditional as is currently the case [49, 50]. This would also move the discourse on from whether or not children should be included, to how this can be achieved.

A further policy implication is to provide clear guidance around issues of ‘voice’. Whilst ‘giving voice’; − that is, documenting the views of children with DLD has become an increasing focus of speech and language therapy research in the last 10 years [32, 37, 38, 51, 52], we know that children with DLD currently have little genuine ‘influence’ in decisions about the services and supports they receive [42]. Lundy proposes a framework which might guide policy makers in this task. She draws a distinction between giving ‘voice’, ‘space’, ‘audience’, and ‘influence’ and argues that all four are necessary if we are to genuinely include children in decisions that affect their lives [49].

### Implications for practice

In addition to the suggested policy changes above, we propose that practitioners need to learn the skills necessary to ‘*listen*’ to children with DLD. Listening, as defined by Clark [53], is not the same as extracting information from the child about an adult-led issue. Different methods of listening have been piloted in research with children with communication previously, such as the use of multiple conversations and multi-modal prompting systems [38, 51]. Practitioners need to be given the opportunity to learn about these techniques, understand their rationale and to use them as part of their everyday interactions with children.

Giving the child genuine ‘influence’ may also require practitioners to be open to thinking and/or working in new ways. This is professionally challenging, requiring enhanced clinical reasoning and problem solving skills, a strong sense of self-efficacy and professional autonomy. In planning SLT services, practitioners would need to be supported to work in such a responsive way.

### Implications for research

The majority of studies in the field of speech and language therapy are focused on establishing the efficacy of procedures to improve the language skills of the child. The views of the children in this study highlight the need for research to guide SLTs and teachers when considering ways of optimising classroom discourse to enable children with DLD to learn. Whilst there is guidance available to ensure a classroom is ‘communication friendly’ [52], there is no coherent theoretical framework currently being applied within the field of speech and language therapy that we know of, which enables us to systematically describe and test out ways of adjusting the rules of class talk for children/young people with DLD. An implication for research, then, is the need to consider different methodologies such as sociological approaches to the study of the classroom.

Finally, it is important to add that the desired services and supports described by these stakeholders are in stark contrast to many of the limited models of speech and language therapy support to schools. For many SLTs, ongoing, carefully-planned dialogue (including the child) with the aim of co-configuring individualised supports, delivered in a way which ensures the child’s inclusion and participation is simply not possible. The findings highlight the need to continue to increase awareness about DLD and to lobby for the necessary resources for SLTs to be able to work in a meaningful way in schools.

### Limitations of the study

This is a descriptive study involving the views of a small number of stakeholders. The findings cannot be said to represent the views of teachers, SLTs, parents or children with DLD in general. Instead, we provide a rich description of the ideal service and supports as described by a group of individuals, carefully chosen because of their particular knowledge and experience in relation to SLT services and supports, in order for us to develop our understanding of collaboration in this context and to propose ways in which it might be facilitated.

## Summary and next steps

We engaged multiple stakeholders in the design of their ideal speech and language therapy service and supports to schools. We found important differences in perspective between the stakeholder groups. Most striking were the unique insights the children brought to the process. They described in detail the many barriers to their achievement, participation and inclusion in school. Further, they were able to describe many practical ways in which these barriers could be addressed and their needs met in an inclusive way;-that is, without setting them apart from their peers.

Up until now, studies of ‘collaboration’ have been limited to understanding what happens between the professionals. We advocate the need, based on our findings, to reframe the process so that the child is given influence in decisions about support in school. In the next phase of our study, we aim to establish consensus about premises that might underpin a model to guide this inclusive approach to collaboration.

## Appendix 1

### Topic guide (parents and professionals)

***Discovery***

Question 1:

Can you think for a minute about a time when you accessed a service and it was really, really ***brilliant.*** Jot down some details: who was there, what was happening, how you felt, what made it so brilliant. Tell the group all about it? Give us as much detail as you can.

***Dream***

Question 2:

Now I want to introduce someone to you (prop). His name is Sean. He is 11 years old. He has difficulty understanding instructions, his reading and spelling is poor and he finds friendships hard. It is hard to make out what he is telling you at times. If you had three wishes for Sean in school, what would they be?

Question 3:

Everything you have ever imagined for Sean has come true. I want you to imagine you are looking in the window of the school/classroom. Tell me what you see. Give us as much detail as you can.

***Design***

Question 4:

Take a minute to look at the flip charts / post-its / your notes/ scribbles, the ideas that we have shared today. Now you need to try to draw a picture of the best speech and language therapy service to school ever. You can use shapes, symbols whatever works for you. You need to draw it rather than use words.

## Appendix 2

### Topic guide (children)

We are going to do three things today.

- ***Remember:*** The best day ever in school (show symbol)
- ***Dream:*** I’m going to give you three wishes about school (show symbol)
- ***Design:*** Then we are going to imagine the wishes have all come true. You can draw the best classroom and school you can imagine (show symbol)

(Place symbols in order, on desk. Refer to symbol at the start of each activity)

***Remember*** (props: story sequence cards)

So first, I’d like you to remember. Can you think of the best day EVER at school? Ok, here is some paper and pens. Can you draw/ write about some things that happened on this day? Here are somethings reminders of things to think about when telling a story (use props: ‘colourful semantics’ cards – what, who, where, when, how feel). Can you tell me about this day?

***Dream*** (props: 3 blank clouds)

Now I am going to give you three wishes about school. Here are your wishes. You can draw on them or write them or talk about them. It is your choice. Can you tell me about your wishes?

***Design*** (props- window, lego man/woman, classroom objects) Now, all your wishes have come true. You are in the best school ever, and all of your wishes have come true. Can you draw one big picture of this school and classroom? (flipchart) You can add things to the classroom. I have some things here that you might find in a classroom you can use or make up your own things?

*Possible prompts to ask during or end:* (Tell me about the children in this classroom? What are they like? Tell me what is happening? How are they feeling? What are you doing in this classroom? How do you feel in this classroom?)

Is there anything else about the best school ever that you can tell me?

## Abbreviations

DLD: Developmental language disorder
IPC: Inter-professional collaboration
SLT: Speech and language therapist
